# The multifunctional regulatory post-proline protease dipeptidyl peptidase 9 and its inhibitors: new opportunities for therapeutics

**DOI:** 10.1007/s00018-025-05719-4

**Published:** 2025-04-28

**Authors:** Jasmine Minh Hang Nguyen, Samuel Zolg, Ruth Geiss-Friedlander, Mark Douglas Gorrell

**Affiliations:** 1 Centenary Institute, Faculty of Medicine and Health, The University of Sydney, Camperdown, NSW 2006 Australia; 2 Center of Biochemistry and Molecular Cell Research, Albert-Ludwigs-Universität, 79104 Freiburg, Germany

**Keywords:** DPP9, DPP4, DNA repair, Inflammation, Inhibitors, Cancer

## Abstract

Dipeptidyl Peptidase 9 (DPP9) is a prolyl amino dipeptidylpeptidase that can cut a post-proline peptide bond at the penultimate position at the N-terminus. By removing N-terminal prolines, this intracellular peptidase acts as an upstream regulator of the N-degron pathway. DPP9 has crucial roles in inflammatory regulation, DNA repair, cellular homeostasis, and cellular proliferation, while its deregulation is linked to cancer and immunological disorders. Currently, there is no fully selective chemical inhibitor and the DPP9 knockout transgenic mouse model is conditional. Mice and humans in which DPP9 catalytic activity is absent die neonatally. DPP9 inhibition for manipulating DPP9 activity in vivo has potential uses and there is rapid progress towards DPP9 selectivity, with 170x selectivity achieved. This review discusses roles of DPP9 in biology and diseases and potential applications of compounds that inhibit DPP9 and its related proteases.

## Introduction

The Dipeptidyl Peptidase 4 (DPP4) gene family is the S9b subgroup of prolyl oligopeptidases, which have a conserved catalytic domain containing serine, aspartate and histidine [1]. This family includes four enzymatic members, DPP4, DPP8, DPP9 and fibroblast activation protein (FAP), and two non-enzymatic members, DPP6 and DPP10.

Over the last decade, members of this family have attracted much attention as potential drug targets because they have roles in numerous physiological and pathophysiological pathways. Notably, DPP4, also known as CD26, contributes to glucose homeostasis by inactivating glucagon-like peptide-1 (GLP-1), an incretin hormone that downregulates glycaemia, and DPP4 inhibitors have been a therapy for managing type 2 diabetes mellitus (T2DM) since 2006 [2–6]. Additionally, the transmembrane bound DPP4 is used as a cell entry receptor by the Severe Acute Respiratory Syndrome Corona Virus (SARS-CoV) that causes Middle East Respiratory Syndrome (MERS) [7]. In contrast, FAP has been gaining a spotlight as a promising radiopharmaceutical theranostic or therapeutic target for cancer [8]. As a canonical marker for an activated fibroblast, FAP is highly expressed in not only fibrotic tissue [9], but also the stroma of many types of solid tumour, predominantly by cancer-associated fibroblasts (CAFs) that support the tumour [10, 11]. To overcome this challenge, chimeric antigen receptor (CAR) T cells targeting FAP^+^ CAFs have been developed and undergone clinical trials [12, 13]. Moreover, FAP inhibitors developed as positron emission tomography / computed tomography (PET/CT) scan are anticipated to be a powerful imaging tool to detect cancer and fibrosis in situ [8, 14, 15]. Lastly, DPP8 and DPP9 (DPP8/9) are becoming increasingly studied as more novel functions emerge. DPP8/9 are involved in a plethora of diverse cellular functions, including regulating cell death, inflammation, DNA repair, B cell signalling, cell proliferation and cell adhesion, and protein degradation in the N-degron pathway. DPP8/9 dysregulation, such as overexpression or deficiency, has been associated with hyperinflammation, cancer, autoimmunity and respiratory diseases.

The protein structures of all four enzymes of the S9b family share an α/β hydrolase domain and an 8-blade β-propeller domain, with the catalytic triad lying at the interface of the two domains [16–18]. DPP8 and DPP9 were the last of the four proteases of this family to be discovered [19, 20]. Both are intracellular amino-dipeptidase that cleave a peptide-bond post proline [21–24]. The *DPP9* gene chromosomal location is 19q13.3 in human and 17.29.2.cM in mouse. In human, two variants of DPP9 have been characterized: DPP9-short (DPP9-S) is 863 amino-acid-long (NCBI designation NP_001371552.1) and is confined in the cytosol, while DPP9-long (DPP9-L) is expressed from an upstream methionine resulting in a protein of 892 residues (NCBI designation NP_001371540.1) that includes a nuclear localization signal near the N-terminus and so can enter the nucleus [20, 23, 25]. DPP9 is highly conserved between human and mouse, sharing 93% sequence homology [23]. Within the DPP4 family, DPP8 shares the highest sequence homology with DPP9, up to 79% amino acid similarity and 61% amino acid identity, which is much greater than DPP4 [20, 23]. DPP9 and DPP8 exhibit high similarity in tertiary protein structures and similar binding dynamics, especially at their catalytic pockets where ligand binding leads to an induced fit conformation and a cooperative binding, which are absent in DPP4 [16]. Specifically, their catalytic domains share up to 92% amino acid identity, causing overlap in substrates [23, 24]. In contrast to FAP and DPP4, DPP8 and DPP9 lack a transmembrane domain and are strictly intracellular.

DPP9 and DPP8 are ubiquitously expressed in human and mice, with the highest DPP9 levels found in spleen, liver, lung, lymph nodes and small intestine [1, 26, 27]. Additionally, DPP9 expression is prominent in leukocytes and epithelial cells [23, 27–29]. DPP9 is abundant and widely distributed in brain [20, 23, 30], and very recently found to have a role in memory [30]. Of the two, DPP9 appears to be more prominent than DPP8, and its silencing is rate limiting for cleavage of proline-containing peptides in cytosolic extracts [22].

In line with the high homology, DPP8 can cut many DPP9 substrates, such as adenylate kinase 2 (AK2), and can interact with NLRP1 and CARD8 [21, 31, 32]. Moreover, synthetic inhibitors almost universally exhibit poor selectivity for one over another protease. Here we will first highlight the current knowledge on the physiological and pathophysiological functions of DPP9, followed by updates on the challenges concerning inhibitor development.

## DPP9 and its role in biology

Discovering DPP9 endogenous natural substrates has been ongoing, with new substrates continually emerging, whereby few have been verified as endogenous substrates, and even fewer have been characterized regarding functional outcomes. DPP9 can cleave AK2, Syk, BRCA2 and RU1_34 − 42_ (VPYGSFKHV) both in vitro and intracellularly [21, 22, 31, 33–39]. Additionally, DPP9 has various binding partners independent of its catalytic activity, notably CARD8, NLRP1, SUMO1, FLNA and Keap1 [34, 36, 40–45]. Importantly, binding of SUMO1 to DPP9 is a key step for the allosteric activation and stabilization of DPP9 enzyme activity [43].

Like other enzymes of the DPP4 family, DPP9 has the rare ability to hydrolyse substrates with a Pro in the P1 position. In vitro assays with recombinant DPP9, and the reporter substrate Gly-Pro-AMC in combination with peptide libraries, allow a systematic analysis of the substrate preference of DPP9. Peptides which bind at a high affinity to the active site of DPP9 inhibit the hydrolysis of Gly-Pro-AMC [46]. Using this approach it was shown that in addition to the Pro in P1 position, DPP8 and DPP9 prefer hydrophobic and aromatic amino acids in P2, while negatively charged residues (aspartic or glutamic acid) in P2 are disfavoured [22]. Discoveries of natural substrates provide additional insights into preferred cleavage sites. These discoveries confirm that DPP9 has a strong preference for post-proline cleavage but is also able to cleave after an Ala at P1, which is penultimate to the N-terminus. In line with the peptide library approach, screens for DPP9 substrates have highlighted substrates with hydrophobic amino acids such as Met, Val, Ala, Thr and Ser occupying P2, followed by Pro [21, 31]. Examples of well characterised DPP9 substrates in vitro that exhibit this characteristic are AK2 (MAP$\:\downarrow\:$S), RU1_34 − 42_ (VP$\:\downarrow\:$YGSFKHV), BRCA2 (MP$\:\downarrow\:$IGSK), nucleobindin-1 (MP$\:\downarrow\:$TSV) and CXCL10 (VP$\:\downarrow\:$LSRT) [21, 22, 31, 33, 34, 38]. Of those, a cellular outcome has been identified for RU1_34 − 42_, BRCA2 and AK2 [22, 33, 34].

Although DPP9-L is known to reside in the nucleus, its biological functions in this compartment remain unclear [25], largely because almost all screens for substrates or interactome utilise the short, cytoplasmic isoform, DPP9-S. A recent study has suggested that DPP9 might have a regulatory role in gene silencing via microRNA [47]. Dipeptidyl peptidase DPF-3, a DPP9 ortholog in *Caenorhabditis elegans*, interacts with the microRNA specific Argonaute protein ALG-1 and regulates Argonaute function [47]. Perhaps mammalian DPP9-L similarly participates in regulating mammalian gene expression.

### Roles of DPP9 in the immune system

#### DPP9 is a suppressor of inflammasome activation and pyroptosis

**Fig. 1:**
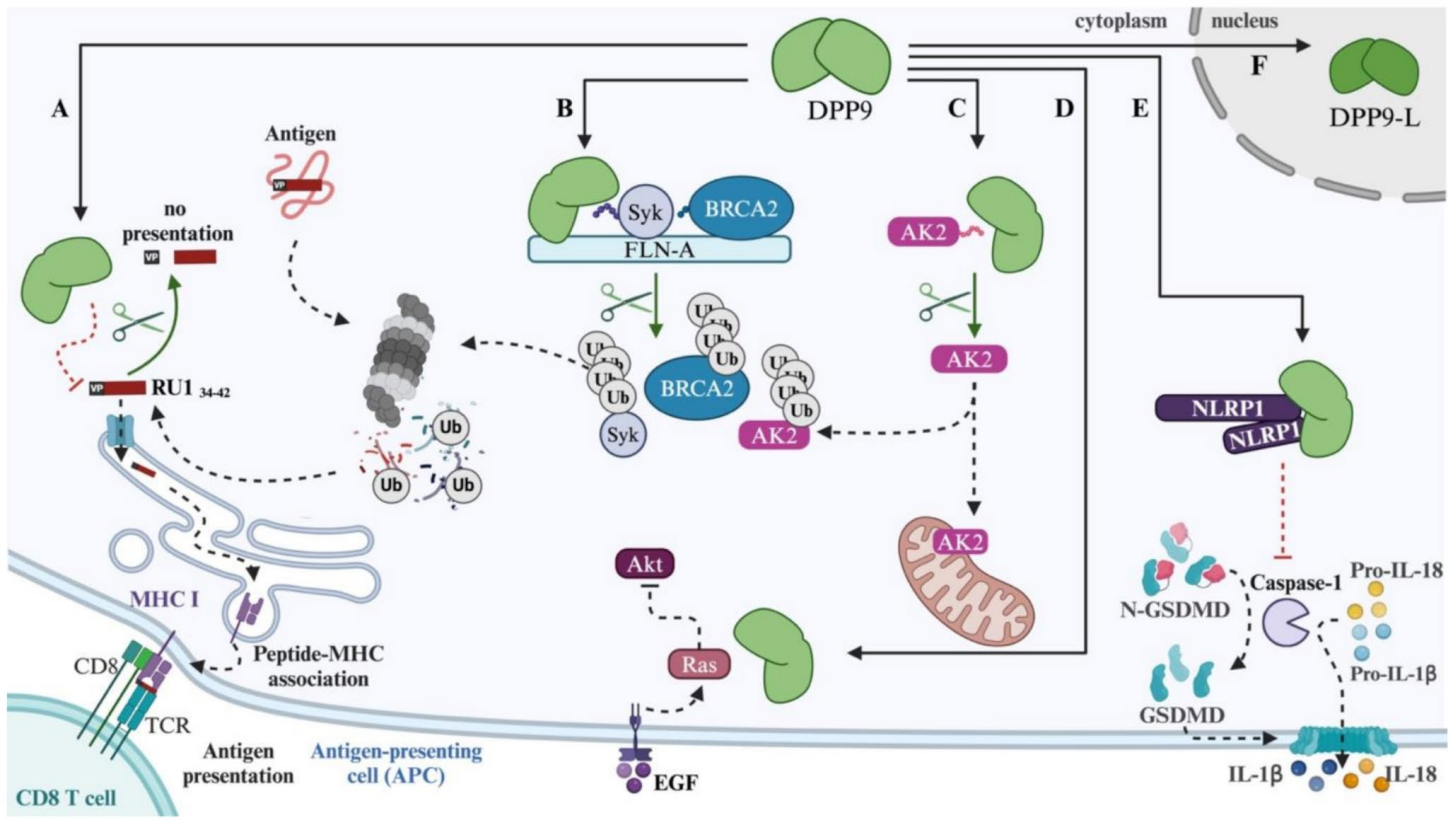
DPP9 in multiple cellular pathways. (**A**) Cleavage of the RU1_34 − 42_ antigen by DPP9 prevents its presentation on the class I major histocompatibility complex (MHC-I) [22]. (**B**, **C**) DPP9 cleaves AK2, BRCA2 and Syk to target these proteins for proteasome degradation via the N-degron pathway [33, 34, 57]. (**C**) The mitochondrial protein AK2 contains an N-terminal DPP9 site. If cleaved by DPP9, AK2 is targeted for proteasome degradation [33]. (**D**) DPP9 regulates a PI3K/Akt pathway by interacting with H-Ras under epidermal growth factor (EGF) stimulation [58]. (**E**) DPP9 represses inflammasome activation by binding to NLRP1. (**F**) DPP9-L (DPP9-long; 892 residues) is a variant of DPP9 with an N-terminal Nuclear Localization Signal [25]. DPP9-S is the canonical 863-residue variant that localizes to the cytoplasm

NLRP1 and CARD8 are protein sensors that can detect pathogen-associated molecular patterns (PAMPs) such as bacterial toxin, viral genetic material and viral protein. NLRP1 is expressed abundantly in human airway epithelial cells, keratinocytes, intestinal epithelial cells and hematopoietic progenitor cells [48–50]. Notably, CARD8 is expressed preferentially in human T cells [51, 52]. Once triggered by PAMPs, NLRP1 and CARD8 auto-hydrolyse and self-oligomerise to form a protein complex, an inflammasome, which then recruits adapter protein apoptosis associated speck-like protein containing a CARD (ASC) and caspase-1 to induce cell death. This results in pore formation on the cell membrane by gasdermin D (GSDMD), causing membrane rupture and the extracellular release of cytokines, primarily interleukin 1-beta (IL-1$\:\beta\:$) and IL-18. This form of cell death is known as pyroptosis. DPP8 and DPP9 can bind to NLRP1 and CARD8 and thereby inhibit inflammasome processing and the downstream activation of caspase 1 (Fig. 1E.) [40–42]. Inhibitors of DPP8/9 can cause the dissociation of these proteins from CARD8 and NLRP1 and thus to activation of pyroptosis [41, 53–55], because binding sites between inflammasome and protease include the catalytic pocket [56].

Initial experiments with DPP9 knock-in mice have shown that DPP9 enzymatic activity is essential for the survival of neonatal mice [59]. Specifically, homozygous *DPP9*^S729A/S729A^ neonates mutated in the catalytic serine (Ser729) survive birth but have 100% lethality rate before weaning [59, 60]. On the other hand, mice are viable if deficient in DPP8, DPP4, FAP or prolyl endopeptidase (PREP) [60]. About one-third of the *DPP9*^S729A/S729A^ homozygous pups can be rescued from death by crossing the parents with NLRP1 knockout (*Nlrp1*^−/−^) or with gene knockout of any of several NLRP1 downstream effectors: *ASC*^*−/−*^, *Gsdmd*^*−/−*^ or *Il-1r*^*−/−*^ [61]. These data indicate that the NLRP1 inflammasome pathway is a major contributor to the observed neonatal lethality in *DPP9*^S729A/S729A^. The rescued pups had significantly shorter bone length and less bodyweight than their wild-type littermates, suggesting a key role for DPP9 in skeletal growth.

Similarly, DPP9 is very important in humans. In 2022, Harapas et al. reported four human DPP9 probands with missense mutation or stop-gained mutation that result in DPP9 loss-of-function [61]. Children born with any of these DPP9 variants share a myriad of symptoms consistent with autoimmune disorders, pancytopenia, anaemia, susceptibility to repeated infections and shorter stature. Severe Coronavirus Disease 2019 (COVID-19) can lead to pulmonary hyperinflammation. Intriguingly, genome-wide association studies (GWAS) have revealed that two single-nucleotide polymorphisms (SNPs) (rs12610495 & rs2109069) in the *DPP9* gene are associated with severe COVID-19 and greater hospitalisation rates [62–64]. Although the mechanism of these SNPs has not been investigated, it is widely speculated that these SNPs produce less DPP9 than wild-type alleles and consequently lead to more NLRP1 activation and aggravation of inflammation in COVID-19.

#### DPP9 in the maturation of antigenic peptide

The major histocompatibility complex class I proteins (MHC-I) are expressed by all cell types and are responsible for presenting short peptides of 8–10 amino-acids in length to antigen recognition receptors on T lymphocytes. These antigenic peptides are derived from the proteolytic cleavage of endogenous intracellular proteins (presentation of self-peptides) and, during infection, of pathogen-derived proteins. The ubiquitin-proteasome system plays a major part in the generation of potential peptides, which undergo further trimming in the cytosol. Peptides enter the ER via the TAP transporter, some undergo further trimming in the ER by two peptidases, ERAP1/2, before they are loaded onto MHC-I molecules. This trimming of peptides both in the cytosol and in the ER determines the repertoire of antigenic peptides that are bound to MHC-I. Several cytosolic peptidases participate in the maturation of antigenic peptides, including leucine aminopeptidase, bleomycin amino peptidase and puromycin amino peptidase. The Geiss-Friedlander team has shown that DPP9 also plays a role in the maturation of peptides for presentation on MHC-I [22]. Specifically, they focused on a peptide originating from the RU1 protein in renal cancer. DPP9 was found to cleave this peptide in vitro, and that inhibition or silencing of DPP9 leads to more presentation of the RU1_34 − 42_ peptide (VPYGSFKHV) to cytotoxic T cells. Thus, modulating DPP9 expression or activity can determine whether the RU1_34–42_ peptide is destroyed or is presented at the cell surface. Silencing of DPP8 has no effect on the presentation of RU1_34 − 42_ [22]. Since DPP9 is rate limiting for cleavage of proline-containing peptides, we speculate that the RU1_34–42_ antigen is one of many proline-containing antigens whose presentation on MHC class I is determined by DPP9. The RU1_34 − 42_ peptide was the first endogenous DPP9 - specific substrate to be determined.

#### DPP9 modulates B cell receptor mediated signalling

The first protein to be characterized as a substrate of DPP9 was Syk, a tyrosine kinase that plays a central role in B cell receptor (BCR) mediated signalling. Syk bridges antigen recognition by the BCR with downstream signalling events, with impacts on B cell proliferation and clonal expansion, as well as B cell differentiation.

Binding of an antigen to its cognate BCR on the plasma membrane leads to clustering of the BCRs, and consequently to phosphorylation of tyrosine residues in transmembrane ITAM (*i*mmunoreceptor *t*yrosine-based *a*ctivation *m*otifs) containing proteins. Phosphorylated ITAMs serve as docking sites for Syk, which binds via two SH2 domains in its amino terminus, resulting in Syk phosphorylation and activation [65]. Subsequently, Syk phosphorylates downstream signalling proteins. DPP9 destabilizes Syk (see below; N-degron pathway), preferentially in its phosphorylated and active form, acting as a negative regulator of Syk signalling, which influences the duration of the response to BCR activation. Additionally, DPP9 targets phosphorylated Syk in resting cells, thereby maintaining low levels of so-called ‘tonic’ signalling. This tonic signalling pathway is independent of antigen binding to the BCR and is crucial for maintaining B cell survival, development, and readiness to respond to antigens [66–68]. Under normal conditions, tonic signalling maintains a constitutive baseline signal in B cells. Its dysregulation can contribute to B cell survival in cancers, as in Burkitt lymphomas, chronic lymphocytic leukemia (CLL) and diffuse large B-cell lymphoma (DLBCL) [69]. Ablation of DPP9 activity can increase tonic signalling and weaken responses to BCR engagement.

### DPP9 in DNA damage repair

DPP9 depleted cells, as well as cells expressing inactive DPP9, are hyper-sensitive to DNA damaging agents, specifically to the presence of DNA double strand breaks (DSBs) [34]. Such lesions are arguably highly toxic if not repaired, as they can lead to DNA deletions, but also to chromosomal rearrangement, a hallmark for cancer. Cells apply two main alternative pathways to repair DSBs. These pathways are non-homologous end joining (NHEJ), which is error prone, and homologous mediated repair (HR), which is mostly error-free. The tumour suppressor BRCA2 is a main player in the HR, by chaperoning multiple copies of the recombinase Rad51 that form stable filaments on the exposed ssDNA. The Rad51 filaments invade sister chromatids and search for homologous sequences that are used as template for repair. DPP9 targets BRCA2 for degradation (see N-degron below), thereby reducing its intracellular concentration. Cells depleted of DPP9 show defects in repair of DSBs by HR, and fewer Rad51 foci, suggesting that DPP9 fine-tunes the concentration of BRCA2 to regulate the stoichiometry with Rad51 that is critical for formation of stable Rad51 filaments and repair [34].

### DPP9 and the N-degron pathway

**Fig. 2:**
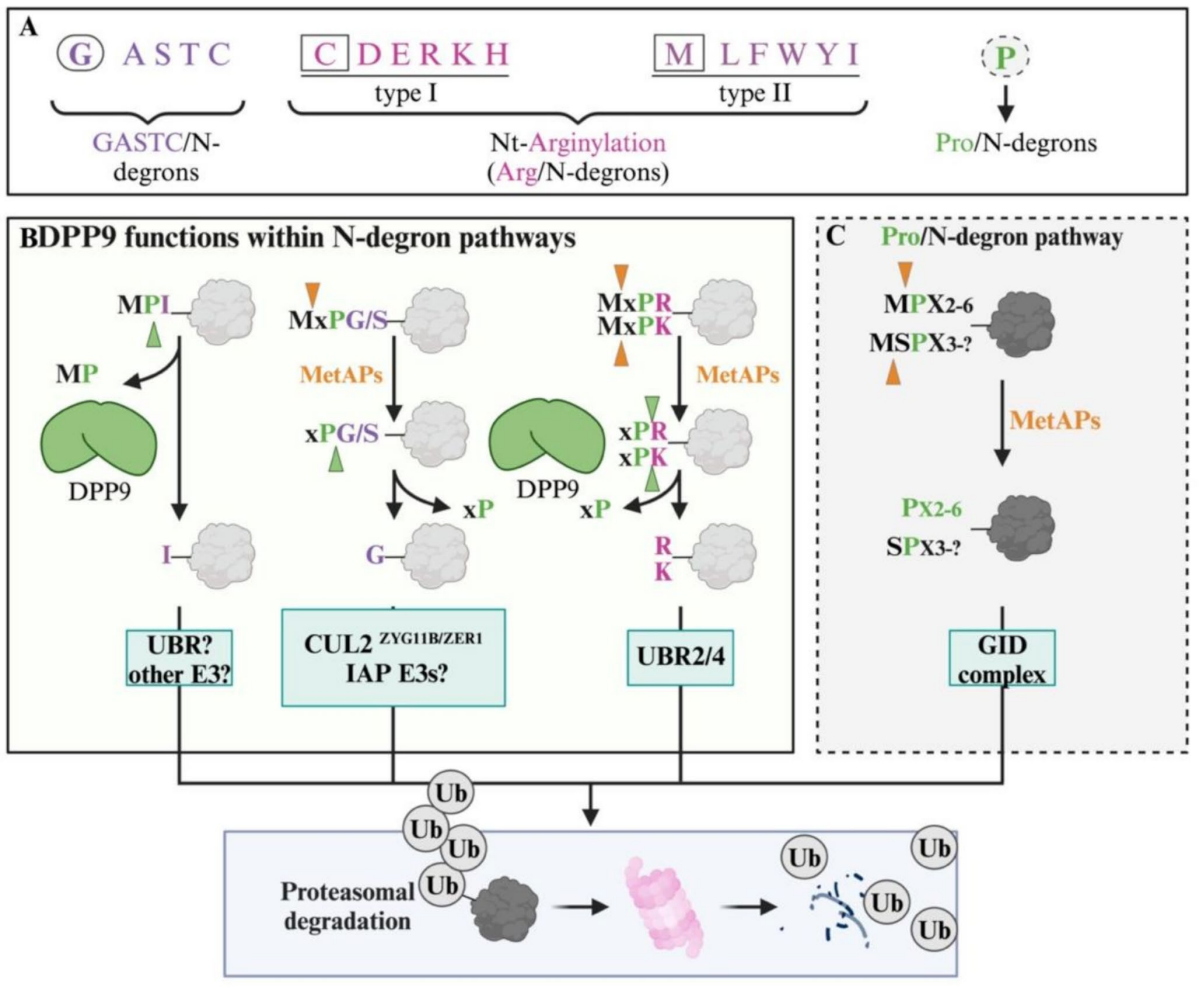
DPP9 is an upstream component of the N-degron pathway. (**A**) An overview of N-degron pathways in mammalian cells. (**B**) Proteins with N-terminal prolines are targeted by DPP9 for the N-degron pathway in mammalian cells. DPP9 removes proline-containing dipeptides from its substrates (Xaa-Pro), thereby exposing cryptic N-degrons. Subsequent ubiquitination by E3 ligases depends on the exposed N-terminal amino acid. (**C**) In yeast, the Pro/N-degron pathway targets proteins that have N-terminal proline, using the GID complex, which is a specialized E3 ligase that specifically recognizes and ubiquitinates such proteins

DPP9 can determine protein stability by removing two amino acids from the N-terminus of its substrates that are then targeted for proteasome mediated degradation [33, 34, 36, 70]. The degradation of proteins based on the nature of their N-terminus is known as the N-degron pathway (Fig. 2, reviewed in [71].

In eukaryotic cells, the N-degron pathway is a branch of the ubiquitin-proteasome system and involves ubiquitin E3 ligases that recognize and bind to N-degrons, meaning destabilizing N-terminal sequences, resulting in the ubiquitination of the substrate and degradation by the 26 S proteasome. This pathway has been extensively studied, with a strong focus on the different E3 ligases, also called N-Recognins, that identify and bind to the N-degrons. The best characterized N-Recognins are Ubr1, Ubr2, Ubr4 and Ubr5, which preferentially bind to proteins with basic (R, K, H) or bulky (F, W, Y, L, I, M) N-terminal residues. Since all proteins are expressed with an initiator methionine, targeting to the N-degron pathway usually requires N-terminal modification, such as acetylation, arginylation or proteolytic processing, for example by methionine aminopeptidases, that act upstream of this pathway to expose N-degrons.

In addition, DPP9 acts as an upstream component of the N-degron pathway. The first link was the finding that DPP9 processes the N-terminus of Syk (Met-Ala↓Ser), leading to its proteasomal degradation [36]. The involvement of DPP9 in this pathway was later demonstrated for mitochondrial proteins such as AK2 (Met-Ala-Pro↓Ser) [33]) and for the tumor suppressor BRCA2 (Met-Pro↓Ile) [34]. In the case of AK2, removal of the initiator methionine must occur prior to processing by DPP9. When cleavage by DPP9 exposes basic amino acids, ubiquitination is carried out by E3 ligases of the N-degron pathway (UBRs) [70]. Processing of BRCA2 and Syk by DPP9 is initiated by a specific signal; the presence of DNA damage or activation of the BCR, respectively, for BRCA2 and Syk. In both cases, interaction of BRCA2 and Syk with DPP9 depends upon the scaffold protein Filamin A which links DPP9 to these substrates [34, 36]. Finally, it should be noted that a Pro/N-degron pathway has been characterized in yeast. This pathway depends upon a multi-subunit E3 ligase complex, the GID complex, that can specifically target proteins with an N-terminal proline [72]. While GID homologs exist in mammalian cells, natural substrates have not been identified. Thus, in mammals DPP9 represents an alternative or parallel pathway to the Pro/N-degron pathway for degradation of proteins with N-terminal proline.

### DPP9 in cell behaviour, proliferation and non-inflammatory cell death

#### DPP9 in cell adhesion and migration

DPP9 has regulatory roles in cellular processes such as cell adhesion, cell migration and cell death. Yu et al. were first to observe that transfected HEK293T cells overexpressing DPP9, but not DPP8, were less adherent and exhibited more cell death [73]. Both DPP8 and DPP9 overexpression reduce cell migration [73]. Notably, similar outcomes were found in cells overexpressing an enzymatically inactive DPP9 mutant, indicating that DPP9 catalytic activity was not required in this in vitro model [73], and that these effects may be due to interaction of DPP9 with yet unknown binding proteins that are not DPP9 substrates. Zhang et al. found that DPP9 overexpression in Huh7 cells localizes prominently to microtubules and also to cell periphery along with adhesion proteins such as integrin-$\:\beta\:$, talin and vinculin following EGF stimulation, which could have promoted cell migration [74]. In the Huh7 liver cancer cell line, DPP9 activity is needed for DPP9 to influence cell migration [74].

Independently, it has been shown that lacking DPP9 causes impaired cell migration and invasion in the A549 and H1299 cell lines [75]. Moreover, overexpressing DPP9 increases cell migration and epithelial-mesenchymal transition markers such as E-cadherin, MUC1 and vimentin [75]. Similar data has been observed in human skin cells [76], where silencing DPP9 gene expression in primary human fibroblasts reduced adherence to collagen, and reduced migration in HaCat cells [76]. Similarly to Zhang et al. [74], DPP9 localized to the inside edge of those cells [76], suggesting that DPP9 is involved in tactile interactions between neighbouring cells and substrata through unknown mechanisms.

#### DPP9 in cell proliferation, apoptosis and ferroptosis

DPP9 is involved in cell proliferation and non-inflammatory cell death such as apoptosis and ferroptosis. Specifically, DPP9 regulates the Akt signalling pathway. Overexpressing DPP9 in the HepG2 cell line inhibits the Akt signalling pathway and involves DPP9 interaction with H-Ras, less proliferation and increases apoptosis following EGF stimulation (Fig. 1F.) [58]. Similarly, overexpressing DPP9 in the Raji human B lymphocyte cell line can increase apoptosis [77]. Concordantly, increased cell proliferation in vitro, and tumour size and mass in vivo, has been observed when DPP9 is silenced in an oral squamous cell carcinoma cell line [78]. However, absence of DPP9 can also result in apoptosis, as seen in A549 cells [75]. DPP9 can have different outcomes in different cancer cell lines, so these phenomena require further study.

DPP9 binding to Keap1 has been implicated in ferroptosis, a kind of cell death involving the accumulation of iron and oxidative stress inside the cell [44]. Keap1 is an E3 ubiquitin ligase that normally tags NRF2, a nuclear transcription factor responsible for regulating reactive oxygen species. DPP9 overexpression in clear renal cancer cells sequesters Keap1 away from NRF2, resulting in an accumulation of intracellular NRF2 and its downstream effectors, thereby increasing resilience against oxidative stress and ferroptosis. By this mechanism, cancer cells can be more resistant to cell death [44]. DPP9 is inactive while it is bound to Keap1, and this is observed for newly synthesized DPP9 [45]. These authors suggested that inactivation of DPP9 by Keap1 may lead to an accumulation of unwanted and mislocalised peptides, an increase in proteotoxic stress and activation of a stress sensor such as NLRP1 [45].

## DPP9 inhibitors

### Pan-DPP inhibitors

**Fig. 3:**
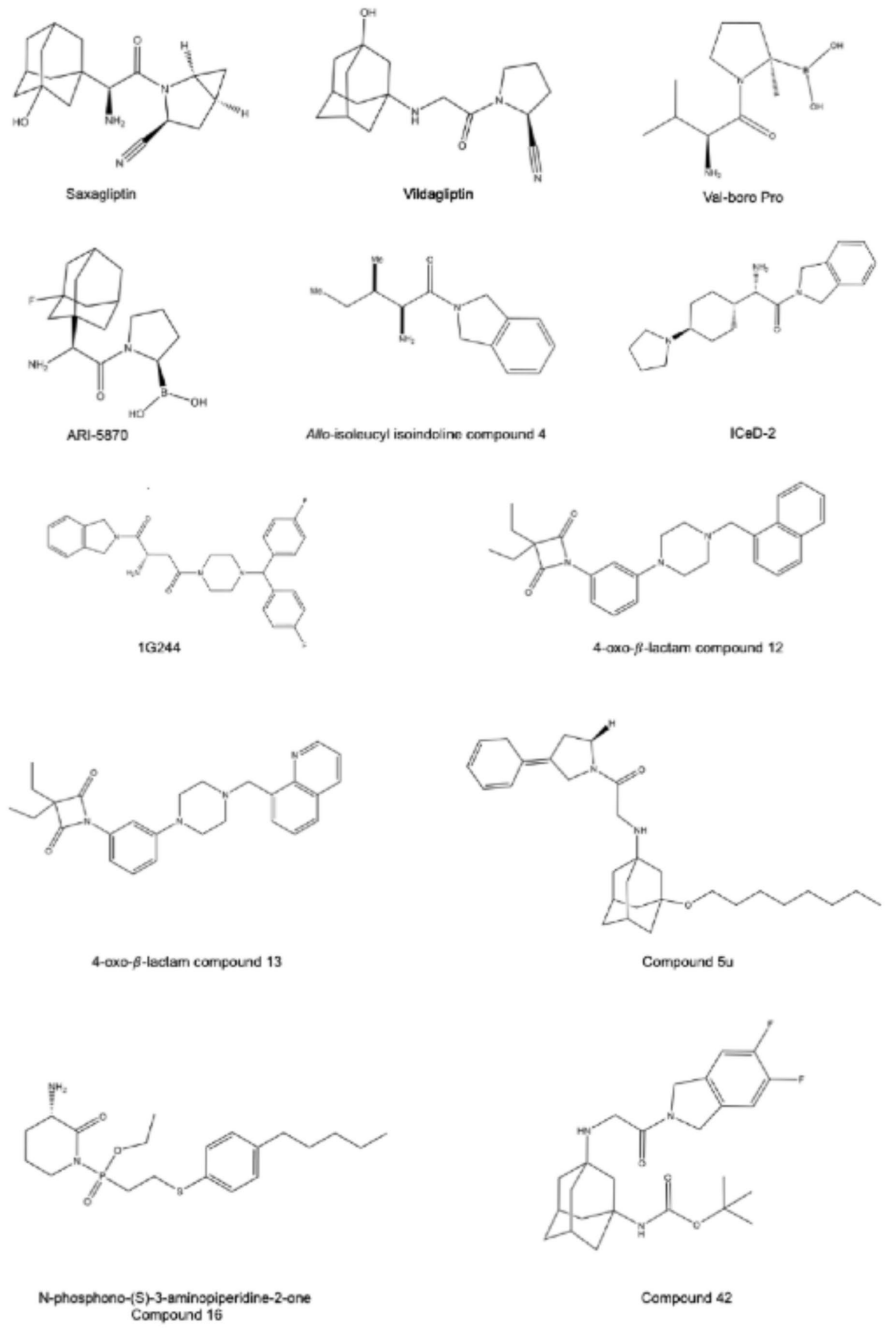
Chemical structures of pan-DPP and DPP8/9 inhibitory compounds that are mentioned in Table 1 (see below)

Prior to the discovery of a DPP9-selective inhibitor, pan-DPP inhibitors, which can inhibit all enzymatic members of the DPP4 family, were commonly used to inhibit DPP9, despite having little selectivity for DPP9 over DPP8. Saxagliptin and vildagliptin (Fig. 3), two potent DPP4 inhibitors (K*i*: 1.3 nM, 13 nM, respectively) can also inhibit DPP8/9 when at high concentrations (Table 1) [79–81]. Notably, vildagliptin is much more selective for DPP9 (K*i*: 0.23µM) and DPP8 (IC50: 2.2µM) and has been modified recently to increase its DPP9 inhibition [82–84].

**Table 1:** Inhibitors used for DPP9 inhibition in vitro and in vivo (rats)

Name	IC_50_ or *K*i (µM)				Selectivity	In vivo observations		
	DPP9	DPP8	DPP4	FAP	DPP9:DPP8	Bioavail-ability (%)	T_1/2_ (hr)	Toxicity
Saxagliptin [79]	*Ki*: 0.098	*Ki*: 0.508	*Ki*: 0.001	Not done.	5x	67	2.5	Alopecia, thrombocytopenia, splenomegaly & death [80]
Vildagliptin [83]	IC_50_: 0.23	IC_50_: 2.2	IC_50_: 0.010	IC_50_: >10	10x	65–69	8.8	None reported [84]
5u [83]	IC_50_: 0.5	IC_50_: 3.3	IC_50_ > 10	IC_50_ > 10	6.6x	Not done [83]		
42 [95]	IC_50_: 0.0034	IC_50_: 0.6	IC_50_: >10	IC_50_: >10	176x	2	0.75	None at 24 h [95]
Val-boroPro [87]	IC_50_: 0.3	IC_50_: 0.01	IC_50_ < 0.004	IC_50_: 0.56	0.4x	Not done.	0.5–2.5	Lethargy, vasocongestion, acute ascites. [88]
ARI-5870 [90]	IC_50_: 0.005	IC_50_: 0.010	IC_50_: 0.009	IC_50_: 0.033	2x	Not done		None after 30-day treatment [90]
1G244 [92]	IC_50_: 0.053	IC_50_: 0.014	IC_50_ > 100	IC_50_ > 100	0.3x	16.5	7	Cyanosis, clonic & tonic convulsions, splenomegaly, thrombocytopenia, reticulocytopenia & death [91]
*Allo*-isoleucyl-isoindoline compound 4 [87]	IC_50_: 0.055	IC_50_: 0.038	IC_50_: 30	IC_50_: >100	0.7x	72	2.5	Thromocytopenia, reticulocytopenia, alopecia, splenomegaly, inflammation, lung histiocytosis & death [87]
SLRFLYEG [96]	*K*i: 0.17	*K*i: 0.147	*K*i > 200	Not done.	0.9x	Not done.		
ICeD-2 [97]	IC_50_: 0.0009	IC_50_: 0.024	IC_50_: 3.5	IC_50_ > 20	27x	68	20	Not done
N-phosphono-(S)-3-aminopiperidine-2-one compound 16 [93]	IC_50_: 298	IC_50_: 14	IC_50_: 16,150	Not done	< 0.04x	Not done		No toxicity in cells
4-Oxo-β-Lactam compound 12 [98]	*K*i > 2	*K*i: 0.095	Not done	Not done	< 0.05x	Not done.		
4-Oxo-β-Lactam compound 13 [98]	*K*i: 0.174	*K*i: 0.0342	Not done	Not done	0.2x	Not done.		

Val-boroPro, also known as PT-100, Talabostat and BXCL701, was initially synthesised as a DPP4 inhibitor for diabetes therapy but was then found to also inhibit DPP8 and DPP9 substantively (Table 1.) (IC_50_: < 4, 4 and 11 nM, respectively) [16, 85–89]. Unfortunately, the toxicity of Val-boroPro at high doses, particularly towards leukocytes due to increased pyroptosis, causes hesitancy for prolonged use in vivo [55, 85]. Recently, ARI-5870, a Val-boroPro analog, was shown to have similar levels of inhibition of DPP4, 8 and 9 (Table 1) (IC50: 9, 10 and 5 nM, respectively) [90]. Mice treated with ARI-5870 orally over 30 days have been found to not lose weight nor display symptoms of toxicity. ARI-5870 appears to have anti-tumour efficacy, reducing subcutaneous tumour size in mice comparable to Val-boroPro [90].

### DPP8/9 selective inhibitors: a juggling act

Most inhibitors developed so far are isoindoline compounds, some of which display excellent selectivity for DPP8/9 over DPP4 and FAP. For example, 1G244 is 7,000 and 2,000 times more selective for DPP8 and 9 over DPP4, with IC_50_ 14 nM, 53 nM and > 100 µM, respectively (Table 1) [91, 92]. 1G244 penetrates cellular membranes in vitro and has a rapid absorbance rate and a 7 h half-life in vivo. However, prolonged exposure (> 24 h) causes toxicity due to off target effects [93], so caution is needed for in vitro studies. Similarly, high doses of 1G244 cause severe toxicity in rats, including cyanosis, thrombocytopenia, splenomegaly and reticulocytopenia, making it unsuitable for use in vivo [91].

An in vivo toxicity study for a DPP8/9 selective compound along with other DPP4 inhibitors reported that *allo*-isoleucyl isoindoline was very selective for DPP8 (IC_50_: 38 nM) and DPP9 (IC_50_: 55 nM) over DPP4 (IC_50_: 30,000 nM) and FAP (IC_50_ > 100,000 nM) (Table 1) [87, 94]. *Allo*-isoleucyl isoindoline has a reported bioavailability of 72% and a half-life of 2.5 h in vivo [87]. A two-week toxicity study in rats showed some toxicities including alopecia, thrombocytopenia, reticulocytopenia and splenomegaly, and bloody diarrhea in dogs at 6 weeks of treatment. Histology results showed some organ inflammation and pulmonary histiocytosis [87]. These data presaged the link between DPP9 and inflammation, which was discovered a decade later [41, 54].

Very recently, two new inhibitors emerged that have greater selectivity for DPP9 versus DPP8. Inducer of Cell Death-2 (ICeD-2) (Fig. 3) was synthesised as part of a phenotypic screening for novel HIV therapeutics and was reported to have 27-fold selectivity for DPP9 over DPP8 (IC_50_: 0.9 & 24 nM, respectively) (Table 1) [95]. Additionally, ICeD-2 displays strong bioavailability, up to 61%, and an excellent half-life of 20 h in vivo in mouse [95]. Independently, Benramdane et al. synthesised several vildagliptin derived compounds, some of which had some selectivity for DPP9 over DPP8. The most promising compound, 5u, had 7-fold selectivity for DPP9 over DPP8 (IC_50_: 0.5 & 3.3 µM) (Table 1) [83]. Both ICeD-2 and 5u are isoindoline-containing compounds, further indicating that such compounds have potential for achieving selectivity within the DPP4 family. Moreover, computer simulations have revealed that isoindoline can stabilise with a unique pair of adjacent glutamates in DPP8 and DPP9, but not in DPP4. These two glutamates are essential for catalysis, are conserved in the $\:\beta\:$-propeller domain of the DPP4 family, and facilitate selectivity for DPP8/9 [18, 24, 83, 96].

A recent breakthrough by Benramdane et al. came with two novel isoindoline-containing vildagliptin-derived compounds that display greatly increased selectivity for DPP9, being 93 and 176 times selective over DPP8 [97]. The most selective inhibitor, compound 42, has IC_50_ 600 nM and 3 nM against DPP8 and DPP9, respectively (Table 1). These inventors have suggested that the interactions of the compounds’ fluorine atom and *tert-*butyl group with the two separate phenyl groups in DPP9s’ S1 and S2 hydrophobic pocket favour the binding conformation. The compound showed poor bioavailability (2%) and a short half-life (< 45 min) in vivo, but there was no observable toxicity in mice. Moreover, compound 42 induces less cell death in vitro, compared to 1G244 [97]. Due to its unprecedented selectivity, the discovery of compound 42 confers new promise into the DPP9 landscape for research and in vivo applications.

Short peptides can act as protease inhibitors, either by binding to regulatory regions on the protease or by acting as non-cleaved substrates that bind at the active site. Such DPP9 inhibitors were developed based on the finding that the *s*mall *u*biquitin like protein *mo*difier, SUMO1, is an allosteric regulator of DPP8/9. The SUMO1 residues that participate in binding to DPP9 involve the sequence SLRFLFEGQRIADNH, and are located to a region in SUMO1 defined as the E67 interacting loop (EIL) [43]. On its own, this EIL peptide binds directly to DPP9 and inhibits its activity. Pilla et al. modified this peptide to several variants, including SLRFLYEG, with an increased affinity to DPP9 (K*i*: 170 nM) and DPP8 (K*i*: 147 nM) and more than a 1,000-fold selectivity compared to DPP4 (K*i*: > 200 µM) [43, 98]. HeLa cells incubated with SLRFLYEG have reduced enzyme activity and increased phosphorylated Akt under EGF stimulation, which is consistent with previous observations from Yao et al. showing that DPP9 affects the EGF pathway [43, 58, 98]. Additional modifications of this peptide produced up to 20-fold selectivity between DPP8 and DPP9 [43].

Yet another class of DPP8/9 inhibitors, 4-oxo-$\:\beta\:$-lactams, were recently described, presenting another exciting alternative to the isoindoline-containing inhibitors. These compounds bind to DPP8 and DPP9 in a covalent manner [99]. The best hit is a naphthalene compound that has 21-fold selectivity for DPP8 over DPP9 (*K*i: 95 nM & >2,000 nM, respectively) owing to a novel interaction that is not observed in DPP9, between the compound and the extended hydrophobic pocket of DPP8 (Table 1). Like 1G244 and Val-boroPro, these compounds can induce pyroptosis [99].

The most recently developed selective inhibitors are N-phosphono-(S)-3-aminopiperidine-2-ones that have been derived from the natural product Sulphostin. Sulphostin is a covalent inhibitor of DPP4/8/9 [93, 100]. Based on structural analysis with DPP8 and DPP9, derivatives of Sulphostin with improved DPP8/9 inhibitory potency have been generated [93]. These inhibitors display enhanced proteome-wide selectivity and display reduced off-target effects. One of those is compound 16 with IC50 values of 14 nM and 298 nM for DPP8 and DPP9, respectively (Table 1). In contrast to 1G244, compound 16 shows no cytotoxicity in cells. Importantly, compound 16 exhibits a DPP8/9 engagement in cells. Moreover, treatment of cells with this compound leads to an increased sensitivity of cells to genotoxic agents, mimicking DPP9 depleted cells [93].

Currently, detecting endogenous DPP8 and DPP9 in situ depends upon antibodies. However, due to the very high homology shared between these two proteins, cross-reactivity is an issue for interpreting DPP9 immunostaining, which is part of a wider issue in biology [101, 102]. To circumvent this issue, Espadinha et al. developed selective DPP8/9 inhibitors conjugated with fluorescent tags [103]. This fluorescent probe is the first to bind into the active enzyme to detect DPP8/9 in live cells in vitro, as a supplementary approach to antibodies. Such a probe detects only active enzyme molecules, and so cannot be used following fixation or used concurrently with a DPP8/9 inhibitor.

## Applications of DPP9-selective inhibitors: breaking new ground for novel therapy

### DPP9 Inhibition as cancer therapy

In cancer therapy, inducing cell death in cancerous cells is one of the crucial steps to promote tumour regression. Many studies have reported that DPP9 is overexpressed in human cancer and cancer cell lines, including ovarian carcinoma, testicular tumours, hepatocellular carcinoma, clear cell renal carcinoma, colorectal cancer, and non-small cell lung cancer, suggesting that inhibiting DPP9 may reduce tumour growth [27, 44, 75, 104–108]. Indeed, lung cancer xenografts with DPP9 silenced grow significantly slower and are less invasive in vivo [75].

Using Val-boroPro, 1G244 and some isoindoline derivatives to inhibit DPP9 induces significant cell death in many human acute myeloid leukemia (AML) cell lines, suggesting a therapeutic potential of DPP9-selective inhibition in AML [109]. Despite being a PAN-inhibitor, currently, Val-boroPro is the most widely used inhibitor of the DPP4 family in cancer research, having been used in several murine cancer models and in clinical trials for malignant solid tumours [110–112]. Val-boroPro, under the synonym BXCL701, has entered a phase I clinical trial for AML [113].

**Fig. 4:**
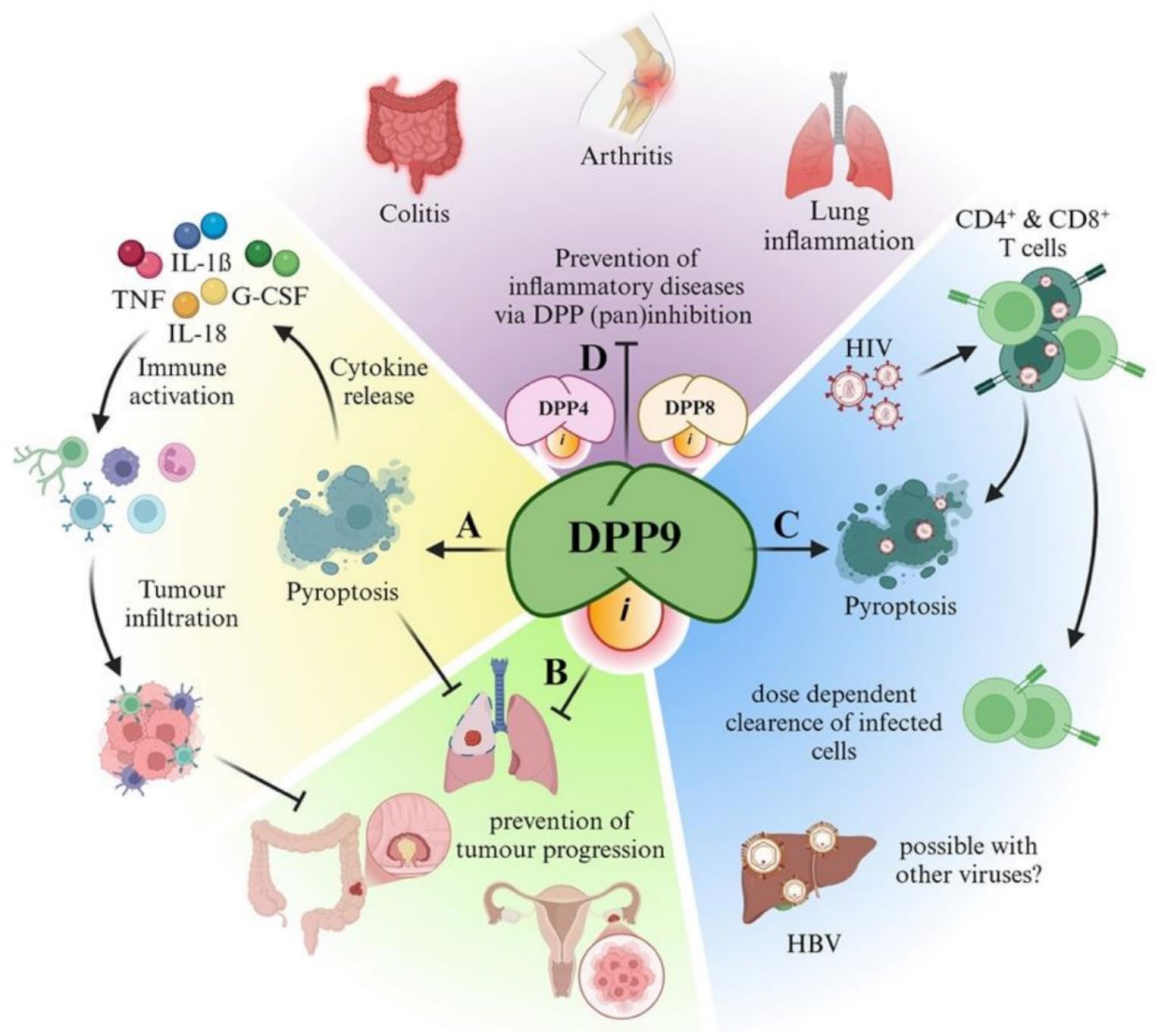
Potential therapeutic applications for DPP9 selective inhibitors. (**A&B**) Anti-tumour action by (**A**) inducing cell death in cancer cells or macrophages, or (**B**) inhibiting tumor progression. (**C**) Anti-viral action in chronic and acute viral infections. (**D**) Anti-inflammatory in chronic inflammation. Inhibitor; *i*

DPP9 inhibition by Val-boroPro is believed to stimulate the production of several cytokines that can act against tumours, such as IL-18, IL-1$\:\beta\:,$ G-CSF, CXCL1 and TNF-$\:\alpha\:$, leading to tumour growth regression in mice (Fig. 4A) [110]. Moreover, Val-boroPro enhances activation of lymph node draining T cells via dendritic cells and T-cell tumour infiltration, hence promoting tumour regression [111], possibly by acting upon DPP9. Val-boroPro can increase the prevalence of cytotoxic T cells and natural killer cells when used in conjunction with an antibody to programmed cell death protein 1 (PD1), develop anti-tumour T-cell memory, and reduce pancreatic ductal adenocarcinoma tumour burden in mice [112]. Moreover, it appears that pyroptosis driven by DPP9 inhibition puts these tumours into a pro-inflammatory state, with increased Th1 cell activation and immune response. A Val-boroPro derivative, ARI-4175, can lower tumour burden in experimental primary liver cancer and rhabdomyosarcoma, but with extended treatment can also increase inflammation and fibrosis [114, 115]. Excessive inflammation caused by pan-DPP inhibition can be ablated by COX2 inhibition [116].

Following the lowering of liver tumour burden by pan-DPP inhibition, Huang et al. knocked out DPP9 only in hepatocytes to find a similar, but muted, outcome in the same liver cancer model, with lowered tumour burden only amongst small tumours. That outcome may indicate that tumour initiation, but not later growth, is retarded by DPP9 loss in hepatocytes [117]. These hepatocyte-DPP9 depleted mice also exhibited lower blood glucose, body mass and adipose tissue mass, so improved energy metabolism is potentially involved in the tumour outcome.

DPP9 inhibition is most prospective as a novel AML therapy. DPP9 inhibition leads to pyroptosis via the NLRP1/CARD8 axis, amongst both lymphocytes and the monocyte-macrophage lineage [51–53, 55]. Most importantly, primary AML cells are more sensitive to Val-boroPro triggered pyroptosis than are other cell lines [109].

Overall, a full understanding for the influence of DPP9 is less clear in breast cancer. For example, DPP9 activity is important for fine-tuning the levels of BRCA2 a prominent tumour suppressor in breast cancer and thus for ensuring correct repair of DNA DSBs by homologous recombination. Down-regulation of DPP9 would lead to accumulation of BRCA2, and thus deregulation of repair. Indeed, high levels of components of this pathway are observed in several cancer types. Depletion of DPP9 sensitizes cells to genotoxic agents such as Olaparib, g-radiation or Mitomycin C. Therefore, combining DPP9 inhibition with Olaparib could be expected to benefit patients (see Sect. 2.2 & 2.3 above; [34]). High DPP9 expression in patients with breast cancer associates with longer survival time, while low DPP9 expression is associated with poorer survival of these patients [34, 118]. These outcomes occur in patients with luminal A subtype, the most common breast cancer subtype, which has the greatest DPP9 gene expression levels. DPP9 expression is the lowest in HER2-enriched and basal-like subtypes, which are more aggressive. A possible explanation for this paradox may be that overall high DPP9 levels are beneficial to the patient as they ensure correct overall DNA repair. Furthermore, DPP9 may also influence cancer progression via additional pathways. For example, long exposure to the DPP8/9 inhibitor 1G244 in MCF7 cells combined with starvation leads to accumulation of lysosomes, with mechanisms possibly involving increased autophagy [119]. Furthermore, DPP9 depletion in a breast cancer mouse model delays tumour onset but ultimately promotes metastasis, by increased levels of the zinc finger transcription factor ZEB1 and accelerated TGF-β1 induced epithelial-to-mesenchymal transition (EMT) [118]. Thus, further investigations are required for discovering and characterizing more DPP9 substrates, as well as investigations in cancer models for a deeper understanding of the different modes in which DPP9 affect cancer progression.

### DPP9 inhibitors in viral infection

Like cancer cells, the idea of inducing cell death in virus infected cells is very attractive, especially for a chronic infection such as HIV. Val-boroPro can induce pyroptosis in CD4^+^ and CD8^+^ lymphocytes [51, 52], leading to its evaluation on HIV-infected T cells (Fig. 4B). Remarkably, both Val-boroPro and ICeD-2 can enhance the clearance of HIV-infected cells in vitro by causing pyroptosis, and both compounds perform even better when combined with an antiviral drug, while having no reported toxicity to healthy T cells at the tested concentrations [95, 120]. Similar findings have been reported using 1G244; at 1 µM it did not affect cell viability in healthy T cells, but caused pyroptosis in HIV-infected CD4^+^ T cells and reduced viral burden [121]. Importantly, CARD8^−/−^ THP-1 cells do not undergo pyroptosis when treated with Val-boroPro, but rapidly die when supplemented with wildtype CARD8 or a mutant CARD8 that is resistant to HIV protease cleavage [121]. The level of activity of CARD8 inflammasome determines post-viral entry cell death in CD4^+^ T cells and this activity is greatly enhanced by DPP9 inhibition [121]. Thus, DPP9 inhibition is beneficial in HIV therapy and potentially in other viral infections. It is tempting to speculate that DPP9 inhibition could alleviate other viral infections in which the virus remains dormant in the hosts’ cells, such as hepatitis B virus (HBV) or herpes simplex virus (HSV).

Coronaviruses cause acute respiratory infections such as COVID-19. Less DPP9 would be expected to exacerbate inflammation in COVID-19, but in situ data suggests otherwise, whereby DPP9 overexpression in peripheral blood has been associated with increased COVID-19 severity [122]. Therefore, it is unclear whether inhibiting DPP9 in vivo would exacerbate or alleviate COVID-19 disease severity.

### DPP9 Inhibition in chronic inflammation

Given the link between DPP9 and the NLRP1 inflammasome pathway, DPP9 inhibition may be beneficial in managing chronic inflammation. The current dogma is that DPP9 sequesters NLRP1 and thereby suppresses inflammation, and DPP9 deficiency or inhibition is pro-inflammatory [40, 41, 54, 61, 114]. However, the antithesis has been observed in several studies, in that DPP9 gene expression and enzyme activity increases in colitis and asthma [123, 124]. Treatment with a pan-DPP or DPP8/9 selective inhibitor can reduce inflammation, eosinophilia and swelling in asthma, colitis, and arthritis (Fig. 4C) [123–127]. Therefore, DPP9 regulation of inflammatory response lacks clarity, including how much DPP9 is required to prevent a pro-inflammatory state.

Finally, several links of DPPs to lung inflammation have been observed, highlighting DPP inhibitors as a potential treatment for lung diseases. Increased gene expression of DPP4, 8 and 9 and increased DPP enzymatic activity following asthma induction occurs in mice, suggesting that pan-DPP inhibition might be protective in asthma [123]. Concordantly, inhibiting DPPs with the pan-DPP inhibitor Val-boroPro results in partial protection against asthma and reduced eosinophil infiltration in mice [125]. Treatment with either Val-boroPro or vildagliptin has been shown to improve outcomes in two separate models of bleomycin-induced pulmonary fibrosis, including less inflammation, improved lung volume and less collagen deposition [126, 128]. Furthermore, human DPP9 gene expression in peripheral blood cells increases during moderate and severe COVID-19 [122].

## Future directions and conclusion

At 25 years since the discoveries of DPP9 and DPP8, the greatest advances have been in discovering that DPP9 gene knockout is neonate lethal, discovery and characterization of substrates and development of novel inhibitors, derivation of 3D crystal structures, deciphering a role in the N-degron pathway of protein regulation, insights into roles in DNA repair, cell death, and interactions of specific inflammasomes with these unique intracellular proteases. There is clear differentiation of these two proteases from DPP4. However, we have little insight into why these two very similar proteases with partially overlapping repertoires of substrates and binding partners, coexist without cross-compensation of expression levels when one is absent. Differences between DPP8 and DPP9 are emerging, and the pace of such discoveries will likely increase as inhibitor selectivity increases. Similarly, characterization of more substrates and binding partners are expected to shed more light into the biological differences between these two enzymes. DPP9 is the only member of its gene family that exhibits lethality when absent from mice, so it may well be the primordial member of the S9b family.

New advances in the design of DPP9-selective inhibitors will allow researchers to further the understanding of DPP9 functions and target it to treat diseases. So far, many of the novel inhibitors in this review, including the most selective compound, have not been tested in vivo, which warrants further investigation regarding their side effects and pharmacokinetics. Currently, there is no DPP9 knockout transgenic mouse line, only conditional knockout, which is a challenge when studying DPP9’s involvement in diverse disease models. Selective inhibitors may overcome that challenge. It is important to seek to differentiate DPP9 from DPP8 by establishing whether any new inhibitor can reproduce previous findings in vitro where a DPP8/9 inhibitor was used. On the other hand, perhaps inhibition of multiple DPPs will synergise such that less selective inhibitors, such as Val-boroPro, that inhibit all the DPP4 family will be shown to be more useful than selective inhibition in cancer therapy.

In conclusion, this review provided a comprehensive overview on DPP9, its role in biology and its chemical and peptide inhibitors. This review discussed potential applications of DPP9-selective inhibitors in treatments of cancer, viral infection, chronic inflammation, and lung diseases.

## Data Availability

No data was generated as part of this paper.
